# Word’s Predictability Can Modulate Semantic Preview Effect in High-Constraint Sentences

**DOI:** 10.3389/fpsyg.2022.849351

**Published:** 2022-03-23

**Authors:** Liling Xu, Sui Liu, Suiping Wang, Dongxia Sun, Nan Li

**Affiliations:** ^1^School of Foreign Studies, South China Normal University, Guangzhou, China; ^2^Philosophy and Social Science Laboratory of Reading and Development in Children and Adolescents (South China Normal University), Ministry of Education, Guangzhou, China; ^3^Center for Language Cognition and Assessment, Guangzhou, China; ^4^School of Psychology, South China Normal University, Guangzhou, China; ^5^Guangdong Country Garden Polytechnic, Qingyuan, China

**Keywords:** semantic, preview, predictability, contextual constraint, eye-tracking

## Abstract

The processing of words in sentence reading is influenced by both information from sentential context (the effect of predictability) and information from previewing upcoming words (the preview effect), but how both effects interact during online reading is not clear. In this study, we tested the interaction of predictability effect and the preview effect in predicting reading processing. In the experiment, sentence constraint was controlled using all high-constraint sentences as materials. We manipulated both the predictability of the target word in the sentence and the semantic relationship between the preview word and the target word as predictors of the semantic preview effect. The results showed that the semantic preview effect was present only when the target word had low-predictability in the sentence but was not observed when the target word had high-predictability in the sentence. The results suggest that contextual information in reading can modulate the pre-activation of words and thus influence whether the preview word has a priming effect. The results of this study provide further evidence that reading comprehension involves an interactive system of processing multiple sources of information at multiple levels.

## Introduction

Reading comprehension is a dynamic process of integrating multiple sources of information. Many studies on reading comprehension have focused on the facilitative effects of two of these sources of information. The reader obtains information through previewing in parafoveal vision. This information has a bottom-up influence on the reading process. The sentence provides information about context, which affects word’s predictability. This information has a top-down influence on the reading process. Previewing and word’s predictability have each been shown to improve reading comprehension. This study tests whether the two sources of information have independent effects on online word processing, or whether the influence of preview information is modulated by word’s predictability. Research on this issue help to reveal how different sources of information can interact to influence the reading process.

Previewing has been shown in a large number of studies to facilitate the subsequent reading process, a phenomenon called the preview effect (see Rayner, 1998, 2009; Schotter et al., 2012, for reviews). Parafoveal processing has been widely documented in eye-tracking experiments using the boundary paradigm developed by Rayner (1975). In this paradigm, the target word is masked during preview, but not masked when it is fixated. The information extracted parafoveally can be studied *via* changing information during the preview. For instance, the target word can be masked by itself (e.g., chair) or by an unrelated word (e.g., light) in the preview. The preview effect is observed when fixations are significantly shorter after unmasked than after masked previews. According to the previous studies, fixation duration gets shorter after previewing valid information about length, shape, phonology, and semantic meaning of a parafoveal word (see Schotter et al., 2012, for a review).

Contextual information in the sentence can facilitate the reading process as well (e.g., Swinney, 1979; Taraban and McClelland, 1988; Albrecht and O’Brien, 1993; Rayner and Well, 1996). Many psycholinguists have proposed that prediction is crucial in sentence comprehension (e.g., Federmeier and Kutas, 1999; Nation et al., 2003; Wicha et al., 2004; Berkum et al., 2005; Delong et al., 2005; Borovsky et al., 2012; Mani and Huettig, 2012). Compared to words with high-predictability, words with low-predictability are fixated for a longer time, have a higher probability of fixation and higher regression rate, and generate stronger brain electrical activity. For example, Rayner and Well (1996) manipulated the predictability of the target word in a sentence and found that high-predictability had a significant positive effect on word processing. Specifically, eye-tracking data showed that the participants’ first fixation duration (FFD) was significantly longer for low-predictable words than for high-predictable words, indicating that the context information provided by the sentence immediately influenced word processing. The context information may promote word processing by quickly activating the semantic features of the word before the word appears. Such results are reported in studies of the Chinese language as well (Rayner et al., 2005).

A fundamental question in language cognition research is whether and how the reading process is affected when there is information available from multiple sources. In this study, we address this question by testing whether previewing of valid semantic information and predictability each has an independent effect, or whether there is an interactive effect, on the reading process. According to the interactive model of language cognition (Marslenwilson and Tyler, 1987), language processing at different levels is an interactive process in which information at a higher level (e.g., context) can influence the processing of information at a lower level (e.g., previewing). Applying this model, we expect that predictability will modulate the semantic preview effect.

The interaction between context and low-level preview information (e.g., length and orthography) in predicting reading processing is well documented. For example, the size of the preview effect due to word length or word orthography is larger when the word is highly expected given the sentence context than when it is not expected (e.g., Balota et al., 1985; Juhasz et al., 2008). High-predictable words yield larger benefits from identical preview than low-predictable words, suggesting that context information affects the early stages of lexical processing (e.g., Balota et al., 1985; Veldre and Andrews, 2018; Staub and Goddard, 2019). Staub and Goddard (2019) interpreted this predictability–preview interaction under a Bayesian reader model. Briefly, the interactions may be due to the predictability effect being eliminated by invalid preview. When there is an invalid preview, early processing of the target takes place foveally but not parafoveally. The perceptual evidence (orthographic information) is simply too strong for prior information (predictability) to have a measurable effect.

In the study of Balota et al. (1985), they investigated how context interacts with high-level preview information, such as semantic previews, to predict reading processing. However, there was less benefit of semantic preview either the target was predictable or unpredictable in the sentence. In recent years, more researchers have begun to focus on this issue (Schotter et al., 2015; Li et al., 2018; Veldre and Andrews, 2018). For example, Veldre and Andrews (2018) looked at semantic (plausibility) preview effects for predictable and unpredictable words. For unpredictable targets, a plausible preview was almost as beneficial as an identical preview. In contrast, for predictable targets, a plausible preview provided smaller and substantially less benefit than an identical preview. However, whereas the preview effect of plausibility can be due to preview and sentence relatedness, it cannot be due to preview and target relatedness. The benefit of preview and target relatedness may be due to a mechanism similar to priming in isolated word recognition, reflecting the strength of connections among words in the semantic network of the mental lexicon. The benefit of preview word plausibility, however, is more related to the reader’s processing of sentence context information. In this case, the benefit of preview word plausibility in interaction with predictability still reflects the effect of the source of sentence information, rather than the interaction between two sources (sentence and lexical preview information).

There are also studies examining how context interacts with the preview effect of preview and target relatedness (Schotter et al., 2015; Li et al., 2018). However, in such studies, contextual effects are tested through sentence constraint rather than word predictability. Constraint and predictability are both relevant for readers’ activities of prediction, but are still quite different. Specifically, the predictability of a word in sentence processing (often called the cloze probability) is measured as a value from 0 to 1, which indicates the proportion of respondents supplying that particular word as a continuation, given the preceding context in an offline norming task. The contextual constraint of a sentence is the degree to which the context establishes an expectation for a particular upcoming word, generally operationally defined as the cloze probability of the highest probability continuation, ranging from 0 to 1 (Kutas and Federmeier, 2011). Thus, the predictability of a word reflects the degree to which that word is activated in the sentence, whereas the constraint of the sentence reflects the degree to which the reader uses the sentence to make a prediction. Because our research concerns word processing, especially how word processing is influenced by information from different sources, predictability is more suitable than constraint for detecting contextual effects on word processing. To the best of our knowledge, few studies have directly tested the predictability of the target and the preview effect of target or preview relatedness.

Although the previous studies on constraints have revealed the role of contextual information in the preview effect of semantic relatedness, the effects of predictability and constraint are often confounded. For example, Li et al. (2018) studied the relationship of sentence constraint and the preview effect of semantic relatedness. The researchers used Chinese sentences as materials. They created one set of sentences with a high contextual constraint that included a target word with high-predictability (e.g., the crowing rooster was still boasting in the courtyard). They created a second set of sentences with low contextual constraint in which the target word had low-predictability (e.g., that rooster caught by Uncle Liu was still crowing). The preview word and the target word were manipulated, so that they were either semantically related (e.g., a preview word “egg” and a target word “rooster”) or unrelated (e.g., a preview word “palace” and a target word “rooster”). Li et al.’s (2018) results are similar to the results of Veldre and Andrews (2018), showing that when the target word was low-predictable in the low-constraint context, the semantically related preview words shortened the fixation duration on the foveal target words. However, this preview benefit was not observed when the target word was high-predictable and in a high-constraint sentence. The results suggested that the semantic preview effect may be influenced by the predictability of the target word. Predictability may decrease the semantic preview effect by increasing the activation of the target word. However, Li et al.’s (2018) study did not separate the contextual constraint of the sentence from the predictability of the target word, as high-constraint sentences only included high-predictable words, and low-constraint sentences only included low-predictable words. For a high-predictable word in a high-constraint sentence, both predictability of word and constraint of sentence may influence the semantic preview effect. First, the target word of high-predictability in the sentence has been pre-activated, and the processing of the pre-activated target word may no longer be easily primed by the preview word, making it difficult to observe the preview effect. Meanwhile, a sentence of high constraint increases readers’ prediction activities, which may occupy cognitive resources and limit the resources that are allocated to the preview process, thus weakening the semantic previewing effect. Therefore, the mechanism of word’s predictability on the semantic preview effect remains to be further investigated.

In the current research, we examined whether the predictability of the target word can influence the semantic preview effect. We explore this question to test our hypothesis that the contextual information would modulate the priming effect of the preview word by increasing the activation strength of the target words. To explore the possibility of this hypothesis, we built on Li et al.’s (2018) experiment by studying the semantic preview effect while controlling the constraints of the sentence (all sentences were high constraint) and manipulating the predictability of target words and the semantic relatedness of the preview word and target word. If the semantic preview effect is similar regardless of the predictability of the target word, it suggests that the semantic preview effect is independent of the predictability of the target word. If the semantic preview effect is different when the target word is high-predictable or low-predictable in the sentence, it suggests that the semantic preview effect on the target word is affected by the predictability of the target word, thus showing a highly parallel semantic processing mechanism of multi-source information. Based on the previous findings, we expect that the benefit of an identical preview may be modulated by target words’ predictability: high-predictable words will produce a larger identical preview effect than low-predictable words.

## Materials and Methods

### Participants

A total of 48 undergraduate students (18 men, 30 women) participated in the experiment. The average age of the participants is 19.6 years. All participants had normal visual acuity or corrected visual acuity.

### Materials

**FIGURE 1:**
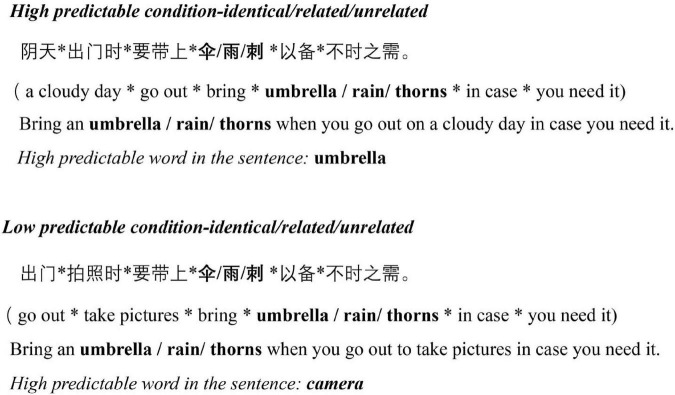
Example of experimental material. In the example, “umbrella” was the target word setting in the experiment, and it was also a preview word under the identical preview condition. The word “rain” was a semantical preview word, which has a sematic association with the target word “umbrella.” The word “thorns” was an unrelated preview word, and there was no relationship between “thorns” and the target word “umbrella.” The target and preview words were embedded in the two kinds of high-constraint sentences with the target word either high-predictable (“Bring an umbrella when you go out on a cloudy day……”) or low-predictable (“Bring an umbrella when you go out to take pictures…….” Note that this is still a high-constraint sentence with the high-predictable word “camera”) in the sentence. In the experiment, when the readers’ fixation located before the boundary, one of the three kinds of preview words “umbrella” or “rain” or “thorns” was presented in the target position. When the reader’s fixation across the boundary, the preview word in the target position immediately turns to the target word “umbrella.”

A total of 84 single-character words were selected as the target words. Another two sets of 84 words that were semantically related or unrelated were chosen as preview words. Thus, there were three types of preview words:(1) identical as the target words, (2) semantically related to the target words, and (3) unrelated to the target words. Word frequency and stroke were matched for three types of preview words. The frequencies for the identical, related, and unrelated previews averaged 289 (standard deviation *SD* = 321), 262 (*SD* = 322), and 259 (*SD* = 276) per million, *F* = 0.24, *p* = 0.783. The number of strokes averaged 8.70 (*SD* = 3.10), 8.75 (*SD* = 2.89), and 8.83 (*SD* = 2.72) for these conditions, *F* = 0.04, *p* = 0.957. The neighborhood size averaged 30 (*SD* = 49), 23 (*SD* = 32), and 22 (*SD* = 37) for these conditions, *F* = 1.15, *p* = 0.315. Targets and previews were embedded in 84 pairs of high-constraint sentences, which were created to make the target words high-predictable or low-predictable in the sentence. The sentence was with 14–19 characters in length and with the target word in the middle of the sentence. There was no punctuation until the end of the sentence. An example sentence and its English translation are shown in Figure 1. The length of the sentence, the position of the target word, and the two words before the target word were the same in both types of sentences. Sentences were presented using the eye-movement contingent boundary technique (Rayner, 1975). In total, 6 counterbalanced material sets were created, each containing 84 experimental sentences. Each condition of the experimental sentences appeared once across the six sets.

A total of 16 undergraduate students participated in the semantic-relatedness rating studies. Participants were asked to rate the semantic relatedness between targets and previews on a 5-point scale (1 = highly unrelated; 5 = highly related). Semantic relatedness for the related preview words (*M* = 4.36, *SD* = 0.33) was significantly higher than the unrelated preview words (*M* = 1.68, *SD* = 0.53), *b* = 2.68, *SE* = 0.07, *t* = 39.50, *p* < 0.001.

A total of 48 participants rated the plausibility of the identical (target), related, and unrelated preview words within each sentence context from the beginning of the sentence up to (and including) the preview word on a 5-point scale (1 = highly implausible; 5 = highly plausible). In the sentence that the target word is high-predictable, the plausibility for the identical, related, and unrelated previews averaged 4.63 (*SD* = 0.52), 1.67 (*SD* = 0.54), and 1.59 (*SD* = 1.09). In the sentence that target word is low-predictable, the plausibility for the identical, related, and unrelated previews averaged 4.53 (*SD* = 0.54), 1.70 (*SD* = 0.52), and 1.63 (*SD* = 0.55). The plausibility of the preview words was not significantly different between sentences with high-predictable target words and sentences with low-predictable target words, *b* = 0.03, *SE* = 0.07, *t* = 0.48, *p* = 0.627. There were neither significant difference between the plausibility of the semantically related preview word and unrelated preview word, *b* = 0.07, *SE* = 0.05, *t* = 1.40, *p* = 0.162, nor its interaction with target word’s predictability, *b* = 0.01, *SE* = 0.10, *t* = 0.10, *p* = 0.920.

To ensure the valid manipulation of the target word’s predictability and the control of sentential constraint, a cloze test was conducted before the experiment. The participants completed each sentence pair from the beginning of the sentence up to but did not include the target word with “The first word that comes to mind.” A number of 60 participants attended the test. The data showed that there was no significant difference in the contextual constraint between the sentence with high-predictable target word (*M* = 0.87, *SD* = 0.11) and the sentence with low-predictable target word (*M* = 0.85, *SD* = 0.11), *b* = 0.02, *SE* = 0.01, *t* = 1.61, *p* = 0.108. In addition, the predictability of target words when it is high-predictable in the sentence (*M* = 0.87, *SD* = 0.11) was significantly higher than when it is low-predictable in the sentence (*M* = 0.04, *SD* = 0.10), *b* = 0.83, *SE* = 0.01, *t* = 53.34, *p* < 0.001.

### Apparatus

SR Eyelink 1,000 eye-tracking system was used to record eye movement at a rate of 1,000 Hz. The movements of the right eye were monitored, in spite of that viewing was binocular. Dell 19-in SVGA monitor displays stimuli with a refresh rate of 150 Hz, and the stimuli took at most 10 ms to complete the display change. The size of each character was 1.0 cm × 1.0 cm, with 0.5 cm between individual characters, and each character was printed in simple Song font.

The participant kept their eyes 57 cm away from the monitor. Each character contains about 1° of visual angle, and the visual angle space between characters is 0.5°. As a result, the spaces before and after each character contain a visual angle of roughly 2°, so that maximizing the likelihood that the target character is in the parafovea when the pre-target character is fixated.

### Procedure

Participants were calibrated with a 3-point procedure and were asked to read each sentence carefully to understand it. After participants completed the five initial practice sentences, each participant read 84 experimental sentences and 30 filler sentences at random. One-third of the sentences were followed by true–false comprehension questions. The experiment lasted for about 30 min.

### Data Analysis

The average comprehension accuracy was 89%. The primary dependent variables (followed those used in Li et al., 2018) were first fixation duration (FFD; the amount of time at which the eyes first fixate on the character, regardless of the number of fixations on the character), gaze duration (GD; the sum of all fixations on a character before moving to another character), and the fixation probability (the probability of fixating the target word on first pass reading) for characters *n* − 1 through character *n* + 1 (relative to target character *n*) as functions of the word’s predictability and preview type. Fixations shorter than 60 ms or longer than 600 m were excluded from the analysis. Trials where the display change occurred during a fixation were excluded. In total, 25% of the data was lost.

We conducted linear mixed effect model (LMM) analyses for durations dependent variables (FFD and GD) and generalized linear mixed models (GLMMs) for binary dependent variables (fixation probability), supplied in the R system for statistical computing (version 4.1.0, Venables et al., 2021). We report fixed effect regression weights (bs), the standard errors of these estimates (SE), the *t*-values (for durations) and *z*-values (for binary dependent variables), as well as *p*-values calculated based on the Satterthwaite approximation for the nominator degrees of freedom (using R package lmerTest). The fixed factors included in the model were target word’s predictability with two levels (high-predictable or low-predictable) and preview type with three levels (identical, semantically related, unrelated). The effects of preview type were specified as two contrasts for estimates of the semantic preview effect (unrelated vs. related preview) and an identical preview effect (unrelated vs. identical preview). All main effects were coded with –0.5 and 0.5 around zero to reflect the difference in the dependent variable between the two-factor levels and also the interaction. The interaction is mathematically equivalent to a main effect because all of them involve contrasts of two conditions against the remaining two conditions (see Shaffer, 1977; Brauer and Judd, 2000, for the reconceptualization of interactions as main effects). Participants and items were included as crossed random factors. The maximum random effects structure that included random intercepts and slopes of factors predictability and preview did not converge, so a simple random effects structure with random slopes removed was used to obtain convergence.

A *post hoc* power analysis was performed using the linear multiple regression method of *t*-tests in the G power software (Mayr et al., 2007). The analysis showed that our experiment had the power of 0.84 to detect a medium effect size (*f*^2^ = 0.15), which has been commonly suggested on preview effects in previous eye-movement studies (e.g., Yang et al., 2012; Li et al., 2018). Our study was therefore well-powered in testing for the preview effect.

## Results

Means and *SD*s of eye movement measures from pre-target character *n* – 1 to post-target character *n* + 1 are shown in Table 1. We report separate analyses of FFD, GD, and fixation probability for each of the three characters.

**TABLE 1:** The mean and standard deviation of eye movement measures in the three regions.

	High-constraint sentence with high-predictable target word			High-constraint sentence with low-predictable target word		
Measure	Identical	Related	Unrelated	Identical	Related	Unrelated
**Pre-target character**						
FFD	237 (41)	244 (46)	242 (47)	228 (42)	247 (44)	248 (41)
Gaze	242 (45)	247 (47)	243 (49)	232 (44)	253 (51)	252 (46)
Fix	0.45 (0.18)	0.43 (0.15)	0.48 (0.22)	0.49 (0.18)	0.54 (0.19)	0.48 (0.18)
**Target character**						
FFD	255 (46)	293 (56)	294 (44)	272 (49)	292 (56)	315 (49)
Gaze	265 (48)	309 (63)	315 (61)	279 (54)	315 (56)	333 (55)
Fix	0.54 (0.20)	0.62 (0.20)	0.63 (0.23)	0.57 (0.18)	0.59 (0.23)	0.63 (0.20)
**Post-target character**						
FFD	266 (41)	268 (45)	279 (42)	268 (47)	290 (52)	277 (58)
Gaze	279 (50)	280 (49)	294 (49)	280 (54)	309 (61)	294 (61)
Fix	0.59 (0.15)	0.56 (0.16)	0.56 (0.19)	0.57 (0.18)	0.59 (0.19)	0.58 (0.16)

### Pre-target Character

The effect of target word’s predictability was significant in fixation probability (*b* = 0.10, *SE* = 0.03, *z* = 2.81, *p* = 0.004). Fixation probability on the pre-target character was lower when target word was high-predictable in the sentence than when target word was low-predictable in the sentence. A hint of a parafoveal-on-foveal interaction of semantic preview effect and predictability was observed on fixation probability (*b* = 0.48, *SE* = 0.18, *z* = 2.57, *p* = 0.010). The result is puzzling, given that the parafoveal-on-foveal semantic preview effect was rarely reported in the previous studies. The *post hoc* test also showed no significant semantic preview effect either in the predictable target word condition (*b* = 0.24, *SE* = 0.13, *z* = 1.78, *p* = 0.075) or in the unpredictable target word condition (*b* = 0.24, *SE* = 0.13, *z* = 1.87, *p* = 0.061). The parafoveal-on-foveal effects may be due to mislocalized fixations (Nuthmann et al., 2005; Drieghe et al., 2008). We did not find in other analysis of significant effect of target word’s predictability and effect of preview type and their interaction.

### Target Character

We found a significant effect of target word’s predictability (FFD, *b* = 12.04, *SE* = 4.05, *t* = 2.97, *p* = 0.002; GD, *b* = 11.41, *SE* = 4.77, *t* = 2.39, *p* = 0.016). Fixation duration of the target word was shorter when it was high-predictable in the sentence than when it was low-predictable in the sentence. The semantic preview effect was significantly interacted with target word’s predictability (FFD, *b* = 19.97, *SE* = 9.73, *t* = 2.05, *p* = 0.040). When the target word was low-predictable in the sentence, FFD in the semantically related preview condition was significantly shorter than that in the unrelated preview condition (FFD, *b* = 24.56, *SE* = 6.88, *t* = 3.57, *p* < 0.001). When the target word was high-predictable in the sentence, however, there was no significant difference in fixation time between the related and unrelated conditions (FFD, *b* = 4.58, *SE* = 6.87, *t* = 0.66, *p* = 0.504). There was no significant effect in the analysis of fixation probability.

We found significant identity preview benefit (FFD, *b* = 43.03, *SE* = 4.98, *t* = 8.62, *p* < 0.001; GD, *b* = 54.81, *SE* = 5.88, *t* = 9.32, *p* < 0.001; fixation probability: *b* = 0.40, *SE* = 0.09, *z* = 4.11, *p* < 0.001). Target word in the identical preview condition was fixated for less time and more likely to be skipped than word in the unrelated preview condition. This identity preview benefit did not significantly interact with target word’s predictability (FFD, *b* = 1.03, *SE* = 9.96, *t* = 0.10, *p* = 0.918; GD, *b* = 4.13, *SE* = 11.74, *t* = 0.35, *p* = 0.725; fixation probability: *b* = 0.12, *SE* = 0.19, *z* = 0.63, *p* = 0.527).

### Post-target Character

The effect of target word’s predictability was significant in gaze duration on the post-target character (FFD, *b* = 8.84, *SE* = 4.14, *t* = 2.13, *p* = 0.033; GD, *b* = 12.91, *SE* = 5.21, *t* = 2.48, *p* = 0.013). Readers fixated for a shorter time on the post-target character when the target word was high-predictable in the sentence than when the target word was low-predictable in the sentence. Meanwhile, the interaction between semantic preview effect and target word’s predictability was significant (FFD, *b* = 27.95, *SE* = 10.21, *t* = 2.73, *p* = 0.006; GD, *b* = 32.72, *SE* = 12.83, *t* = 2.47, *p* = 0.013). However, contrary to the semantic preview effect observed on the target character, the fixation time on the post-target character was longer in the semantically related preview condition than that in the unrelated preview condition when the target word was low-predictable in the sentence (FFD, *b* = 14.57, *SE* = 7.01, *t* = 2.08, *p* = 0.037; GD, *b* = 17.87, *SE* = 8.80, *t* = 2.03, *p* = 0.042). When the target word was high-predictable in the sentence, there was no significant difference between the related and unrelated preview conditions (FFD, *b* = 13.37, *SE* = 7.39, *t* = 1.80, *p* = 0.071; GD, *b* = 13.84, *SE* = 9.31, *t* = 1.49, *p* = 0.137). We also found identity preview benefit (GD, *b* = 15.22, *SE* = 6.43, *t* = 2.36, *p* = 0.018) on the post-target character, and the effect did not significantly interact with target word’s predictability (GD, *b* = 1.00, *SE* = 12.83, *t* = 0.08, *p* = 0.937). There was no significant effect of fixation probability in any analysis.

## Discussion

We investigated whether the semantic preview effect is modulated by the predictability of the target word when the contextual constraint was controlled. When the target word was high-predictable in a high-constraint sentence, we did not observe any semantic preview benefit on the processing of target words. However, when the target word was low-predictable in a high-constraint sentence, semantic preview effects were found. Specifically, a semantically related preview word facilitated the processing of target words first and then hindered the processing of target words later. The results suggest that the semantic preview effect is influenced by the degree of activation of the target words in a high-constraint context.

First, the lack of a semantic preview effect when the target word was high-predictable in a high-constraint sentence is consistent with Li et al.’s (2018) results. More importantly, a new finding in our study is that we observed the semantic preview effects in the high-constraint sentence when the target word was low-predictable. It indicates that the word’s predictability can independently influence the semantic preview effect in the high-constraint sentence. The results supported our hypothesis that rich contextual information making words predictable can decrease the semantic preview effect. This may be due to that the predictability increasing the activation strength of the target words and decreasing the priming effect from a semantically related preview word, thus demonstrating an early interactive mechanism of parallel processing multi-source information.

The interaction between the semantic preview effect and predictability that we observed is consistent with Veldre and Andrews’s (2018) results. However, it should be noted that the semantic preview effect in our study was tested by preview and target relatedness, whereas the semantic preview effect in Veldre and Andrews (2018) was tested by preview plausibility. The two may involve different semantic processes. Actually, the benefit of preview and target semantic relatedness is not as consistently reported in English as it is in some other language systems, such as Chinese. It seems that the semantic preview effect observed in English may be due more to the preview effect of plausibility (Veldre and Andrews, 2016). In Chinese, both semantic relatedness preview effects and plausibility preview effects are observed (e.g., Yan et al., 2009; Yang et al., 2012, 2014). The semantic preview effect can be derived from the semantics of both sentences and words. These differences in semantic preview effect between English and Chinese may implicate cross-language differences in the way that semantic information is coded in preview. However, it seems that no matter how semantic information is coded in preview cross-language, its preview effect is consistently modulated by the predictability of targets.

We did not observe an interaction between the identity preview effect and word predictability in this Chinese-speaking sample. That is, invalid preview words did not reduce the predictability effect. This result is inconsistent with the results of some previous studies with English language readers (e.g., Balota et al., 1985; Veldre and Andrews, 2018; Staub and Goddard, 2019). However, the current results are consistent with those reported by Li et al. (2018) in a sample of Chinese language readers. That is, Li et al. (2018) also did not observe an interaction between the identity preview effect and predictability (or constraint). This suggests that Chinese readers can still retain the effect of predictability even when the preview word is invalid. The different pattern of context effects between Chinese and English may be due to the fact that word processing in Chinese tends to be highly context-dependent. For example, Chinese readers have to rely on contextual information to segment words online (Chen, 1996, 1999), thus making contextual effects stronger in Chinese reading.

Compared with the semantic preview effect observed for low-predictable words in the low-constraint sentence in Li et al. (2018), the semantic preview effect that is observed in our study for low-predictable words in the high-constraint sentence was more complicated. First, like Li et al. (2018), the processing of low-predictable words was facilitated by semantically related preview words, with shorter fixation times on the target words. However, a late semantic preview cost was also observed on low-predictable words in a high-constraint sentence, with increased fixation times on the post-target character. This preview cost may be related to the inhibition of low-predictable targets in the high-constraint context. According to the previous ERP studies, the high-constraint context inhibits the semantic processing of low-predictable words in the later stage of word processing (Federmeier et al., 2007; Kuperberg, 2007). Our study shows that the inhibition of low-predictable words in the high-constraint context is more prominent after a semantically related preview word. One possibility is that the semantic priming from the related preview enhanced the activation of the low-predictable target, which led to a greater inhibition of the low-predictable words. An alternative explanation has to do with how difficult it is to integrate mild and strong semantic violations. Integrating mild semantic violations has been shown to produce higher P600 amplitude than integrating strong semantic violations due to unrelated words (e.g., Yun et al., 2020), suggesting that mild semantic violations may be more difficult to be integrated and need more re-analysis or repair processes. The semantic relationship with a low-predictable target might decrease the semantic violation of the preview word; the mild semantic violation may produce the integration difficulty later. Future studies could extend the current results and investigate these possibilities.

It should be noted that although we found that the semantic preview effect was modulated by the target word’s predictability, all sentences were highly constrained. As mentioned above, contextual constraints and word predictability may each independently influence the semantic preview effect. Future studies on the independent and interactive effects of sentence constraints and word predictability on the preview effect would greatly improve our understanding of the mechanism of the semantic preview process.

The results of this study help us to understand the role of readers’ predictions in the preview effect. It suggests that the semantic preview effect can be modulated by the degree of the pre-activation of target words in a high-constraint context, which indicates that contextual information in the reading process could influence readers’ degree of word processing in real-time, further changing the priming effect of the preview word. The results provide evidence that language comprehension is a highly interactive system of multi-level sources of information for word processing.

## Conclusion

In our experiment, we found that the target word’s predictability can modulate the semantic preview effect with the control of contextual constraint. The results underline the important role of predication in the preview processing of words during sentence comprehension.

## Data Availability Statement

The original contributions presented in the study are included in the article/supplementary material, further inquiries can be directed to the corresponding author.

## Ethics Statement

The studies involving human participants were reviewed and approved by South China Normal University. The patients/participants provided their written informed consent to participate in this study.

## Author Contributions

LX, NL, and SW: conceptualization and writing–review and editing. LX, SL, and DS: investigation and methodology. LX and NL: writing–original draft. All authors contributed to the article and approved the submitted version.

## Conflict of Interest

The authors declare that the research was conducted in the absence of any commercial or financial relationships that could be construed as a potential conflict of interest.

## Publisher’s Note

All claims expressed in this article are solely those of the authors and do not necessarily represent those of their affiliated organizations, or those of the publisher, the editors and the reviewers. Any product that may be evaluated in this article, or claim that may be made by its manufacturer, is not guaranteed or endorsed by the publisher.
